# The Mirror of Dentistry

**Published:** 1853-01

**Authors:** 


*The Mirror of Dentistry. A Review of the Present State of the Dental Profession.* By J. W. Davenport, *Surgeon Dentist, Hull, England.* Seeley's, London.

To be a beautiful quack, in any department of the medical sciences, requires an extraordinary amount of tact. The pretender must be a cunning, shrewd, hypocritical knave—full of bombast, and by words, to expose such pests becomes an honorable calling, and any man who will fearlessly step forward and make the attempt, does society at large, and the honorable members of his profession good service. The author states in his preface that in writing the following pages his object has been to lay before the inquiring public an outline of the present state of dentistry—a plain statement of facts, unbiassed by any contemporaneous opinions; and to check, as far as possible, the most shameless quackery and imposture, by the diffusion of information, in a popular form, long wanted among all persons interested in an art so indispensable to health and good society.

In carrying out those intentions to any extent, we fear the author will not succeed, he has like many others, fixed the *price* of his publication beyond the reach of the ordinary reading public, consequently will cramp the circulation. In these free trade times the public require cheap literature as much as cheap bread. After treating upon the subjects of mechanism of the mouth, dental surgery, he observes, "There is perhaps no part of the dental art, in which greater mistakes have been made and delusions practiced, than in that termed stopping the teeth. The most preposterous statements may constantly be seen, respecting cements, which are said, when applied, almost to rival the teeth in appearance and durability—such pretensions carry on the face of them, the stamp of deception."

Gold, he says, "is far the best, where it can be applied with safety, but in all cases it will not remain."

We regret to see the following observations: "Where the cavity of a tooth is so large that the walls are too thin to bear the pressure necessary for the insertion of the gold foil, the amalgam of silver, or of palladium may be used, and a paste formed by its mixture with quicksilver. This cement will remain useful and permanent for years, when other stoppings would not. The only objection to this, that it changes color and disfigures the front teeth, and therefore it is better adapted for the molars, all stoppings in the shape of an amalgam change color in the mouth. Vegetable cements, such as gutta percha, &c., have been tested for this purpose, but they will not answer, or resist the heat of the mouth; for temporary purposes nothing can be better."

There has been more labor bestowed in endeavoring to discover an *easy method of filling teeth* with amalgams than any other portion of the dental art, and after all, the best amalgams now in use are found to contract and discolor, their indiscriminate use for all classes of teeth, have done irreparable injury to dentistry in England. As a substitute for these, Hill's vegetable stoppings has completely superseded their use and are not open to the objections to which the best amalgams are liable.

The author in speaking of the effects of mercury upon the teeth, its abuse and injurious tendency upon the constitution, says, "Mercury being a mineral, it is often administered where an animal poison in the system exists; and if not watched in its action, its effects are fatal. This remedial agent may do good in the hands of a skillful practitioner, but in the possession of those empirics, who glut for gold, not caring for the life of their patient, it is a viper's bite, and almost as deadly in its effects. Quackery, under any form, is repulsive and despicable, but when covered by the plea of doing good to the human system, either in a medical or artificial point of view, is tenfold more so."

In the truth of the above quotation we perfectly agree, but we cannot reconcile to our minds, if mercury indiscriminately administered internally, so as to produce these effects upon the constitution, in ever so small an extent, why the author should recommend so strongly the filling of teeth with a compound containing all the elements which, when placed in the mouth, will produce similar results? we must leave him to answer in a future edition of his work.

Mr. Davenport is well read in his professional literature, and quite alive to the means that would elevate his profession, strongly advocating combination, unity of purpose and the establishment of a dental college, but alas, he knows not the paltry jealousies that influences men's minds and actions. He says, "the effect of such a college as the one I would now advocate, would be to secure to the public, in a great measure, fair dealing, which is alone the true secret of success in a professional career. At the same time it would retard the progress of no one. If it would, then I would cease to advocate it. It is not to procure the emoluments which may be derivable from the profession, for the benefit of the few, that I seek to have a professional title, so to speak, established; but it is to abolish the trade of the imposter and scoundrel; and to do away with the extortion of quackery in any guise. There are individuals whose tastes and talents may have led them to obtain for themselves a thorough knowledge of some, even of the most learned professions, even though they have been in a very low state in society. And so in dentistry. If a man does but make himself thoroughly conversant with all the principles and practice of the art, then he is our equal. It is so in all professions, and how many are there in the present day, who have shaken off the trammels of mediocrity, and fought their way, step by step, through the most difficult and adverse circumstances, to the highest pinnacle of honor. Men who have shown in the brilliancy of their intellectual greatness, and have written their thoughts in language that will make them admired and beloved by future generations." These are the sentiments publicly and spiritedly put forth, and which ought to be echoed by all popular writers, and with the exception of the recommendation of amalgams, &c., we think the author has done the profession and the public a substantial and lasting benefit. R.

